# A Study on the Intuitive Design of Target Search Tasks under Time and Information Pressure

**DOI:** 10.3390/brainsci12111464

**Published:** 2022-10-28

**Authors:** Jue Qu, Hao Guo, Wei Wang, Sina Dang, Haiping Liu

**Affiliations:** Air and Missile Defense College, Air Force Engineering University, Xi’an 710051, China

**Keywords:** EEG, eye movements, visual search, intuitive design, image schema, stereotypes, expertise

## Abstract

In modern warfare, operators of radar equipment are confronted with a large amount of information in a short period of time that they must process to make decisions. Such conditions often lead to human error, resulting in the loss of the best operational opportunities and putting themselves at a disadvantage. To address this issue, in this paper, we presents three intuitive designs for radar display interface icons, namely image schema, stereotypes, and expert intuition. Based on event-related potential techniques and eye-movement techniques, a modified sample delay matching task experimental paradigm is used to investigate the advantages and mechanisms of three intuitionistic designs with varying time pressures and numbers of tasks. The experimental results showed: (1) When representing the attributes of a target, image schema are most suitable for expressing the motion attributes of the target, such as speed and height, whereas stereotypes are most suitable for expressing fixed attributes, such as target threat. (2) Tasks with high time pressure are more difficult, leading to higher error rates; the reaction time of a single task should be no less than 4000 ms. (3) When multiple attributes of the target need to be observed at the same time, the same type of expression should be used. (4) Rational use of color to represent the target attributes can effectively reduce the cognitive load of people searching for the target.

## 1. Introduction

In the field of military operations, in the face of increasingly complex and rich information, it is difficult to achieve intuitive and effective communication using digital interfaces between humans and computers. For example, the general aircraft, missile, and other targets in the detection range of radar surveillance usually appear for a very short time. In the context of a saturation attack, war fighters need to read a large amount of information, resulting in a high cognitive load of the visual search task, in addition to high time pressure. In such situations, it is difficult for operators to ensure that timely and accurate decisions can be made, and information miscalculation or omission may occur. Therefore, it is an urgent task harmonize the operation interface of accusation systems with the cognitive characteristics of humans under time and information pressure to ensure the efficiency of the visual search task.

Intuitive interactive perception is an important concept representing a development direction in the field of ergonomics with respect to how to improve the interactive efficiency of man-machine interfaces and to ensure rapid interaction between people and devices [1]. However, in current human-computer interactions, artificial intelligence systems are not capable of understanding intuitive physics, even compared with young children [2]. Therefore, human intuition is usually studied in human-computer interactions. Human intuition constitutes the ability to understand things immediately without conscious reasoning. This ability can improve the efficiency of our personal and professional lives [3]. Perception is unconscious and automatic in the process of information processing, enabling the processing of large amounts of information in a short time without logical thinking and slow logical reasoning [4,5]. Many studies have shown that intuitive judgment can be proficient as long as the environment provides effective situational clues and the opportunity to learn clues [6].

Based on the characteristics of intuitive perception, many scholars have applied intuition to human–computer interaction design to reduce the cost of learning new products and the cognitive load of people under complex conditions. For example, Glöckner [7] found that the average decision time for a person in a situation requiring the synthesis of complex information was 20.5 s when the individual responded after conscious analysis, compared to 1.53 s in the same situation when the response was made intuitively. Therefore, the author concluded that under time pressure, people can retrieve information quickly using automatic strategies if the interface information is presented in an intuitive form. The man–machine interfaces of digital cameras, remote controllers, and other devices were studied by Blackler [8,9,10,11], with experimental results proving that intuitive interfaces designed based on familiar features can reduce cognitive processing time and effectively improve operation speed and accuracy, leading to the conclusion that the higher the degree of intuition of the interface, the more reliable the operation of people under pressure or emergency conditions.

## 2. Theoretical Background and Research Questions

### 2.1. How Intuition Arises

The origins of intuition has been explored by many scholars. Patterson [6] suggested that “behind intuition is the analysis of experience”, i.e., people’s intuitive perception is based on the accumulation of experience. Bowers, Regehr and Balthzard suggested that intuition refers to a kind of foreknowledge of coherent information, such as the pattern, meaning, and structure of cognitive objects, before a conscious representation of the objects has been formed [12]. Rosenblatt and Thickstun believe that intuition occurs when the current perceptual pattern is unconsciously matched with the perceptual pattern previously stored in the memory system [13]. Although scholars have different understandings of intuition, they agree that intuition is derived from prior knowledge. However, Hurtienne [14] posits that transcendental knowledge may originate from multiple sources, which can be classified on a continuum, from innate knowledge and knowledge from specific interactions with the physical world (sensory movement) and culture to professional fields; therefore human intuition can also be classified on a continuum. The knowledge continuum and intuitive continuum are specifically related as follows:

(1) Innate knowledge “acquired” through genetic activation or prenatal developmental stages, comprising reflexive or instinctive behavior;
(2) Sensorimotor knowledge consisting of general knowledge acquired early in childhood and used continuously through interaction with the world. For example, when children learn about gravity, they establish the concept of speed and animation. Imagery is present at this level of knowledge;
(3) Knowledge of the culture in which an individual lives. For example, what is known in Western cultural groups is not necessarily equivalent to what is known in Eastern cultures (e.g., appropriate colors at funerals). This corresponds to the stereotypes of people; and
(4) Expertise acquired in one’s profession, from which expert intuition is often derived.

In this paper, we use the levels of knowledge described above as a basis to investigate the different levels of the intuition continuum. Because body reflexes are relatively limited with respect to interface interaction, only imagery schemas, stereotypes, and expert intuition are investigated.

### 2.2. Neurophysiological Studies of Intuition

With the development of neuroscience techniques enabling the recording of brain activity, Wen-Jui Kuo [15] published an article in Science in 2009 revealing, for the first time, the neural mechanisms of intuitive decision making from a neuroscientific perspective. fMRI imaging of two types of experimental tasks completely distinguishing between intuitive and inferential decision-making patterns revealed that the middle frontal gyrus, inferior parietal lobule, and precuneus were more active in solving inferential than intuitive tasks. Gretchen reported on the work of Bechara, Damasio, and others [16], who included patients with ventral prefrontal damage and healthy controls as subjects and simulated a gaming scenario with four decks of cards, two of which were categorized as good, whereas the other two were bad, and asked the subjects to choose from the four decks of cards. The results showed that unconscious emotional signals were responsive to the decision before the conscious decision, which was actually the emotional component of intuition. They also suggested that the ventral prefrontal lobe may store intuitive emotional information from long-term memory at the time of punishment. Whether a neural feature that rapidly encodes the motivational salience of events, i.e., P300, can predict intuitive prosocial motivation was explored by Carlson et al. [17] using event-related potentials (ERPs). The results of the study showed that high P300 amplitudes can indicate intuitive prosocial motivation during prosocial decision making. Yaozhong Liu [18] explored the electrophysiological mechanisms of intuitive decision making using event-related potentials (ERPs) and a modified stimulus flow paradigm and found that the neural mechanisms of intuitive decision making are more complex than those of analytical decision making, and that the neural mechanisms of these two types of decisions may be distinct.

Among the techniques available to record brain activity, fMRI (functional magnetic resonance imaging) and ERP (event-related potentials) are the most mature and widely used [19]. fMRI is used to record brain activity by imaging changes in magnetic induction. This method provides high spatial resolution; however, whole-brain scanning is slow, resulting in low temporal resolution. ERP is used to record changes in brain voltage with high temporal resolution but is affected by cranial and scalp impedance, resulting in low spatial resolution. Both techniques objectively reflect stimulus events in the brain causing activity in the corresponding brain regions; the former relies on changes in cerebral blood flow to trigger induced magnetic fields, whereas the latter relies on neuronal discharges to generate micropotentials. Both techniques have associated advantages and disadvantages and are therefore not interchangeable. fMRI devices are bulky, with slow imaging speeds and are therefore not applicable in the context of the present study. Therefore, ERP was used to investigate intuition in this study.

Various experimental paradigms have been proposed to measure cognitive load, such as the n-back paradigm [20] and the modified sample delay matching task experimental paradigm. This n-back paradigm involves a continuous processing task and is mainly used to study working memory capacity. Such experiments can manipulate the load of working memory by controlling the size of n to examine the processing mechanisms of working memory under different memory loads. Researchers also often use n-back tasks to measure working memory capacity. They can also be used to induce brain loads. In the field of traffic driving, this paradigm has been used as a driving subtask to manipulate the difficulty of experiments by controlling the size of n to control the level of evoked brain load. In the modified sample delay matching task experimental paradigm, a subject is first presented with one sample stimulus, followed by a delayed time interval and two or more comparison stimuli. Combined with the actual interface search task flow, the improved experimental paradigm for the sample delay matching task is used in this paper. This paradigm was applied by Yafeng Niu to the study of iconic memory under varying time pressures, and the results were analyzed with respect to the P300 waveforms [21].

### 2.3. Research Questions

In summary, most research on intuitive design conducted to date has focused on improving the overall ease of use and ease of learning of the product without considering the impact of time and information pressure. Therefore, previous research has not targeted specific interaction tasks. Accordingly, in the present study, we investigated different intuitive interface design approaches from both time pressure and information pressure perspectives for the target search task of radar interfaces. Specific research questions include:

(1) What is the effect of time and information pressure on human cognitive load?
(2) Which of the three intuitive design approaches (imagery illustration, stereotypes, and expert knowledge) is most appropriate to express target attributes, especially under time and information pressure?
(3) Do the advantages of each of the three intuitive design approaches change under time and information pressure; if so, why?

## 3. Materials and Methods

### 3.1. Participants

Sixty participants (30 male and 30 female) were recruited from the Air Force Engineering University to participate in this experiment. The participants were aged 19–25 years, right-handed, and without cognitive operational impairment. None of the participants were found to have central nervous system disorders at the time of physical examination, nor EEG abnormalities, and all had bare eye vision or corrected vision above 5.0. Subjects should have sufficient rest before the start of the experiment to avoid intense reactions and maintain emotional stability. Furthermore, hair should be washed and cut appropriately short; no medication should be taken within 24 h before the start of the experiment; and alcohol, tea, and coffee should be prohibited to avoid affecting the reliability of the measurement of relevant physiological parameters during the experiment. Participants were provided with complete information about the experimental protocol, signed an informed consent form, and were compensated for their participation in the study.

### 3.2. Stimulative Materials

A radar search interface is mainly used to display information such as the position and speed of flying targets. In this study, the radar search interface aircraft icons and missile icons were intuitively designed to express three types of information: speed, altitude, and threat level.

#### 3.2.1. Design of Speed Display

The design of the target velocity expression is shown in Table 1. The image graph targets were designed according to the method described in [22] based on the list of image graph patterns (as shown in Table 2). According to human intuition, the brighter the object is, the higher the energy it emits; for example, the tail flame of an aircraft flying at high speed tends to be brighter, so the brighter an icon representing an aircraft, the higher the speed in the imagery icon experiment. Stereotype targets were designed according to the method described in [23]; a questionnaire survey was distributed to the subjects from our university to screen the stereotype targets to fit the subject group. According to the results of the screening, the stereotypes of the selected subject group were as follows: a flat aircraft wing represents low speed, a slightly angled wing represents medium speed, and an integrated wing represents high speed; a missile without a wing represents the highest speed (e.g., intercontinental missiles usually travel at speeds above Mach 10), a small wing represents medium speed (e.g., air-to-air missiles usually travel at speeds of no more than Mach 5), and a large, flat wing represents low speed (e.g., cruise missiles usually travel at speeds not exceeding the speed of sound). The expert intuition group target material was designed with reference to a radar interface icons; a box icon represents an aircraft, a triangle icon represents a missile, and number of yellow dots traces represents the speed of the target (more dot traces indicated higher speeds).

#### 3.2.2. Design of Height Display

The design of the height of the target expression is shown in Table 3. According to the common sense of near–large and far–small in daily life, the size of icons was used to indicate the height of the target in the imagery icon group, i.e., a larger icon indicates lower height. The letters with icons in the stereotype group represent the height of the target: H for a high-altitude target, M for a medium-altitude target, and L for a low-altitude target. The number of horizontal lines in the expert intuition group represents the height of the target; more horizontal lines represent a higher height.

#### 3.2.3. Design of Threat Degree Display

The design of the target threat level expression is shown in Table 4. The imagery illustration group uses the degree of picture filling to indicate the threat level of the target; the more blue filling, the higher the threat level of the target. The color in the stereotype group represents the threat level of the target; red represents a high threat level, yellow represents a medium threat level, and green represents a low threat level. In the expert knowledge group, the degree of encirclement of the icon represents the threat level; the higher the degree of encirclement, the higher the threat level.

### 3.3. Tasks and Processes

Two kinds of experiments were conducted: time pressure and information pressure experiments. Three design methods were implemented: image schema, stereotype, and expert intuition. The specific design interface is shown in Table 5. The number, position, and information of all icons is the same in the three interfaces.

The subjects were divided into three groups—image schema, stereotype, and expert knowledge—with 20 people in each group. Subjects in the professional knowledge group were trained in radar operation. Each participant wore an electrode cap, sat in a comfortable chair in a room with soft light and sound insulation, and stared at the center of the screen. The distance between participants’ eyes and the screen was about 100 cm, and the viewing angle of horizontal and vertical pictures was within 2 cm. The eye movement equipment was turned on and calibrated. All the experiments were carried out from 10:00 to 12:00 in the morning, and 60 repetitions were carried out under medium pressure to ensure the reliability of the experiment.

**Figure 1:**
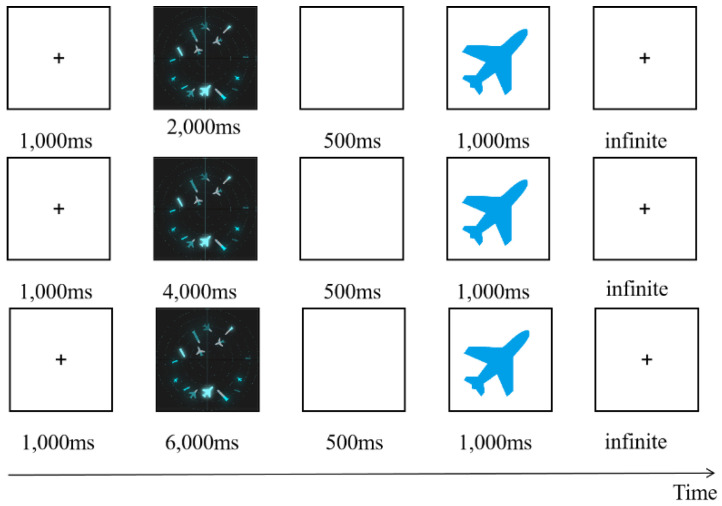
Time pressure experimental process.

The experimental process under pressure is shown in Figure 1. In order to familiarize participants with the task, they were allowed to practice before the experiment. In the first step, the subjects read the instructions, and were then asked to look at the “+”in the center of the screen for 1000 ms. Then, the task interface shown in Figure 1 appeared, with varying interface presentation times (2000 ms, 4000 ms, and 6000 ms), followed by a blank screen for 500 ms. In the second step, a sample icon with single piece of information was randomly presented to the subjects (the single piece of information of the sample icon is shown in Table 6), and the subjects were asked to recall whether the sample icon had appeared in the previous interface. In step 3, participants were asked make judgments and reactions after a blank screen appeared: “If the icon appears, press the ‘A’ key, and if it doesn’t, press the ‘L’key”. In the last step, participants were asked to look at the “+”in the center of the screen for 1000 ms; then, the next round of tasks began.

**Figure 2:**
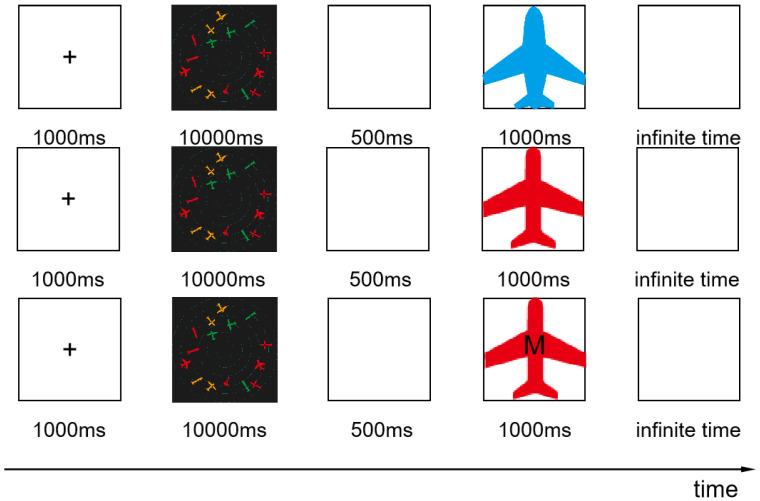
Information pressure experimental process.

The experimental process under information pressure is shown in Figure 2. In order to familiarize participants with the task, they were allowed to practice before the experiment. In the first step, the subjects read the instructions and were then asked to look at the “+” in the center of the screen for 1000 ms. Then, the task interface appeared for 30,000 ms, followed by a blank screen for 500 ms. In the second step, a sample icon was randomly presented to the subjects. The information pressure was controlled by adjusting the amount of information (single information, double information, or three pieces of information) expressed by the sample icon. Subjects were asked to recall whether the sample icon appeared in the previous interface. In step 3, participants were asked to provide judgments and reactions after a blank screen appeared: “If the icon appears, press the ‘A’ key, and if it doesn’t, press the ‘L’ key”. In the last step, participants were asked to look at the “+” in the center of the screen for 1000 ms; then, the next round of tasks began. In the two experiments, the number and types of icons on the interfaces, as well as the information and order represented by the sample icons presented to the subjects, were the same for all three groups.

### 3.4. Electroencephalography Data Collection and Analysis

The equipment used to collect experimental data included a Neuroscan-NuAmps EEG instrument with a maximum sampling frequency of 1000 Hz, which was used to collect EEG signals during the experiment, and SIM-RED eye movement instrument to collect eye movement characteristics during the experiment.

Stimuli were presented using E-prime, and EEG signals are presented using 64 Ag/silver chloride electrode caps based on the extended international 10–20 system. A reference electrode was placed in the binaural prominence. The grounding electrode was located at the midpoint between the FZ and FPZ. Throughout the experiment, the electrode impedance was kept below 5 kΩ, the sampling rate was 1000 Hz, the reference ground electrode was GND and REF, and the signal from the bilateral mastoid electrodes was referenced. In this experiment, we focused only on changes in EEG signals of the subjects after the stimulus was presented. EEG recordings were started when the blank screen was presented, and EEG data were recorded after the stimulus appearance was presented, each of which needed to be superimposed and averaged separately. Eye movement data were recorded using BeGaze.

## 4. Results

### 4.1. Analysis of Experimental Data under Time Pressure Conditions

#### 4.1.1. Behavioral Data Analysis

Behavioral data, including icon memory recognition accuracy (ACC) and response time (RT), were recorded based on key answers provided by the participants. ACC is the ratio between the number of icons correctly recognized and the total number of icons used. RT is the time between seeing the target stimulus and recognition (as indicated by “A” or “L”).

**Figure 3:**
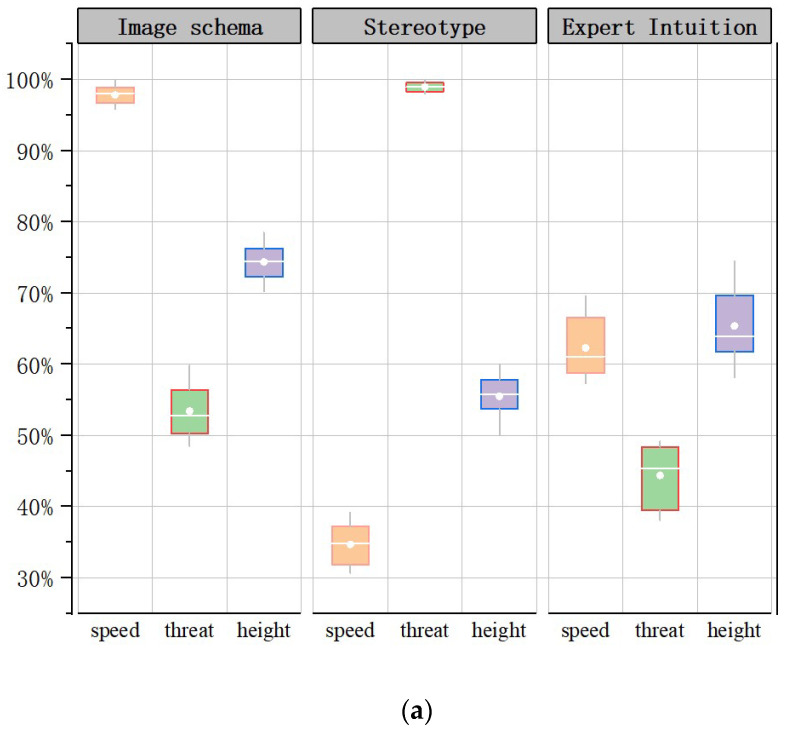

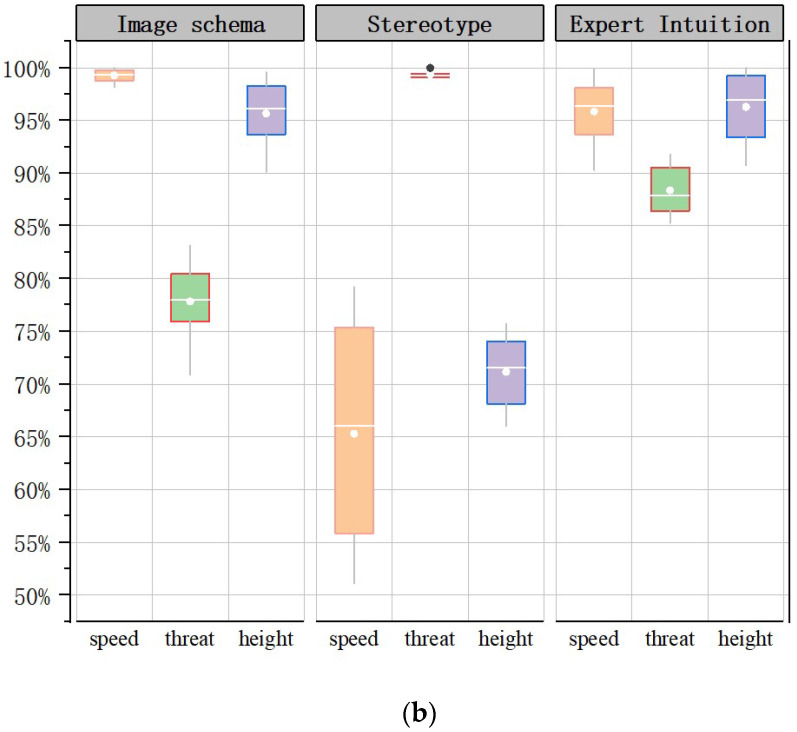
Accuracy box line chart. (**a**) 2000 ms ACC; (**b**) 4000 ms ACC.

Kruskal–Wallis tests were conducted to investigate correctness between different types of tasks under the same time conditions. The correctness of subjects completing the three types of tasks under 2000 ms time pressure differed significantly between the imagery schema group, the stereotype group, and the expert intuition group (*p* = 0.024 < 0.05; *p* = 0.001 < 0.05; *p* = 0.012 < 0.05). Post hoc multiple comparison corrections were performed, and the results of the two comparisons differed significantly. The correct response rates under the 2000 ms and 4000 ms time pressure conditions are shown in the Figure 3 (subjects completed the task with an accuracy close to 100% under 6000 ms time pressure). In the imagery icon group, subjects under 2000 ms pressure were achieved a higher correctness rate on the search target speed task than the threat level and height tasks, with an average correct rate of 98%, 54.6%, and 73%, respectively; subjects under 4000 ms time pressure achieved a 93% correctness rate in completing the target height search task and a 95% correct rate under 6000 ms time pressure in completing the threat level search.

To further explain the perceptual mechanism of different intuitions for the same time conditions and the same type of task as above, Kruskal-Wallis tests were conducted on the correct rates between different groups. (1) Under 2000 ms time pressure, the correct rates of the three groups of subjects completing the speed, height, and threat search tasks differed significantly (*p* = 0.020 < 0.05; *p* = 0.023 < 0.05. *p* = 0.018 < 0.05). Post hoc multiple comparison corrections showed significant differences in the results of the two comparisons. (2) Under 4000 ms time pressure, there was no significant difference between the correct rate of the imagery icon group and the expert knowledge group when subjects completed the speed search task, and there was a significant difference relative to the stereotype group (*p* = 0.007 < 0.05); the correct rate of the three groups when subjects completed the threat level search task differed significantly (*p* = 0.003 < 0.05); the correct rate of the imagery icon group when subjects completed the height search task differed significantly different from the two other groups, whereas the there was no significant difference between the correct rate of the expert knowledge group and the stereotype group (*p* = 0.012 < 0.05), and the results of the two comparisons were significantly different when corrected for multiple comparisons. (3) There was no difference in the accuracy of the three groups of subjects under 6000 ms time pressure in completing each task.

Under 2000 ms time pressure, subjects in the intentional icon group completed the speed search task significantly more correctly than the other two groups; subjects in the stereotyped impression group completed the threat level search task significantly more correctly than the other two groups. Under 4000 ms time pressure, both the intentional icon group and the expert knowledge group achieved an accuracy rate of more than 90% on the speed and height tasks, whereas the stereotyped impression group achieved lower accuracy. All three groups of subjects achieved more than 90% accuracy under 6000 ms time pressure.

**Figure 4:**
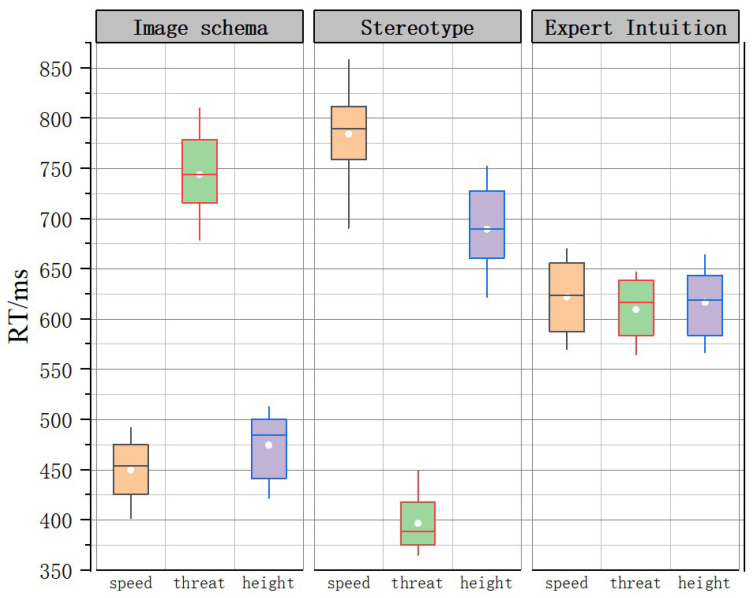
Reaction time data box line diagram.

ANOVA was performed on the reaction times between different types of tasks under the same time conditions (reaction time data are shown in Figure 4). Reaction times differed significantly among subjects in the 2000 ms imagery schema group and the stereotyped impression group for the three types of tasks (*p* = 0.024 < 0.05; *p* = 0.001 < 0.05), and post hoc multiple comparison correction resulted in significant differences between the two comparisons. No significant differences were found between the 4000 ms and 6000 ms time-stressed imagery, stereotype, and expert intuition groups in terms of response time to the three types of tasks.

#### 4.1.2. Eye Movement Data Analysis

**Figure 5:**
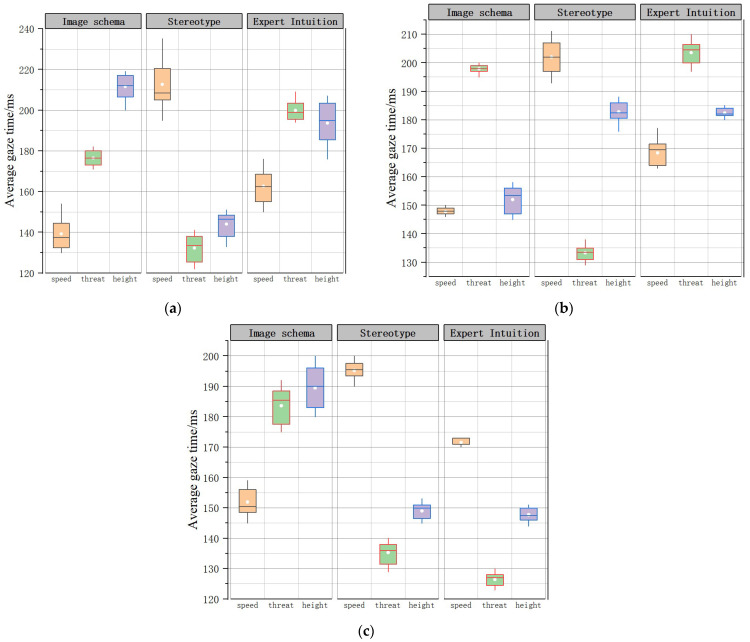
Average gaze time. (**a**) 2000 ms; (**b**) 4000 ms; (**c**) 6000 ms.

**Figure 6:**
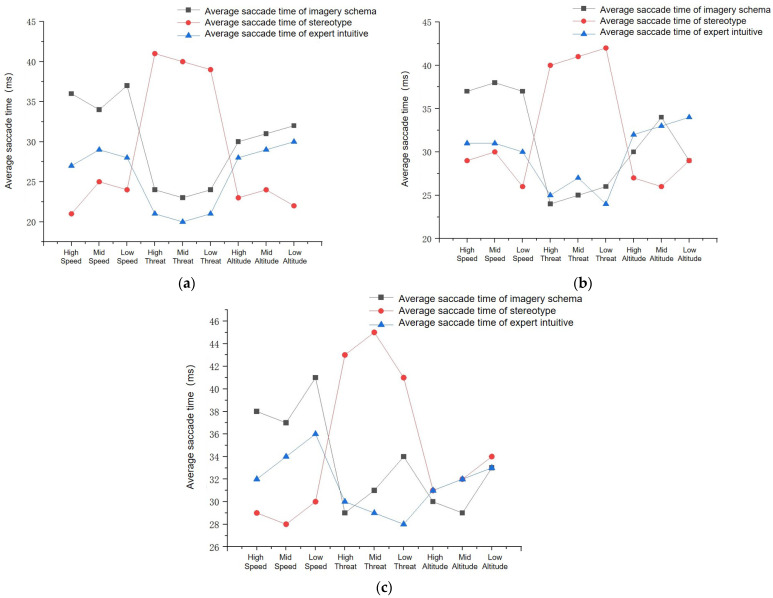
Average eye jump time. (**a**) 2000 ms; (**b**) 4000 ms; (**c**) 6000 ms.

According to the experimental procedure, BeGaze software was used to record the eye movement data of subjects searching for the target. First, the mean gaze time and mean eye jump time within a trial of experimental subjects in the same group under varying time pressures were analyzed separately by ANOVA; the results showed that the mean gaze time and mean eye jump amplitude did not significantly differ (F= 9.63, *p* = 0.027; F = 1.705, *p* = 0.154; F = 4.334, *p* = 0.073). Therefore, only different tasks within each group under the same time pressure were analyzed. The mean gaze time and mean eye jump time are shown in Figure 5 and Figure 6, respectively.

The mean gaze time represents the subjects’ search process time. Figure 5a shows that the mean gaze time of the stereotype impression group under 2000 ms pressure was short in the threat and height tasks, and the mean gaze time of the imagery icon group was shortest in the speed task. Figure 5b shows that the mean gaze time of the stereotype impression group under 4000 ms time pressure was short in the threat task, and the mean gaze time of the imagery icon group was shortest in the speed and height search tasks. Figure 5c shows that the mean gaze time of the stereotype impression group under 6000 ms time pressure was short in the threat and height tasks, and the mean gaze time of the imagery icon group and the stereotype impression group were similar in the speed task.

Table 6 shows that the mean eye jump time was longer in the threat level task for the stereotype group and longer in the speed task for the imagery icon group under the three time pressure conditions.

In summary, the search process for the threat and height tasks was short in the stereotype group, and the speed search process was shortest in the imagery icon group, with subjects able to obtain more information in the pre-eye jump annotations; therefore, these tasks were less difficult, consistent with the results of the behavioral data analysis.

**Figure 7:**
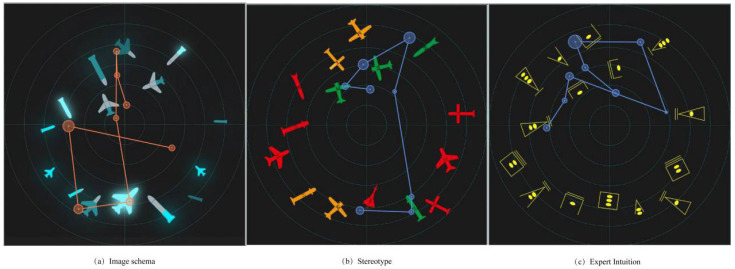
Sweeping path diagram.

A comparison of the scanned path diagrams of different groups (Figure 7) revealed that the subjects’ scanned paths always passed the brightest and largest target in the diagram in each task experiment of the imagery icon group, whereas this phenomenon was not observed in the scanned path diagrams of the other groups, indicating that the high-brightness icons attracted more attention of the subjects.

#### 4.1.3. EEG Data Analysis

Numerous studies have shown that the P300 and P200 ERP components of working and short-term memory in the prefrontal and combined parietal, occipital, and temporal cortices [24,25,26,27,28,29,30,31] are important for iconic memory. P300 is caused by subjective probability, stimulus importance, decision-making ability, decision confidence, stimulus uncertainty, attention, memory, and emotion. The peak latency of the P300 ERP component occurs at approximately 250 to 750 ms, representing neuronal activity during cognition. Therefore, in this study, we analyzed the P300 component to investigate intuitive cognitive mechanisms under temporal and information stress. We selected seven electrodes in the parieto-occipitotemporal joint cortex, including left the P3, CPz, Pz, and PO3; the midline of POz; and the right P4 and PO4.

**Figure 8:**
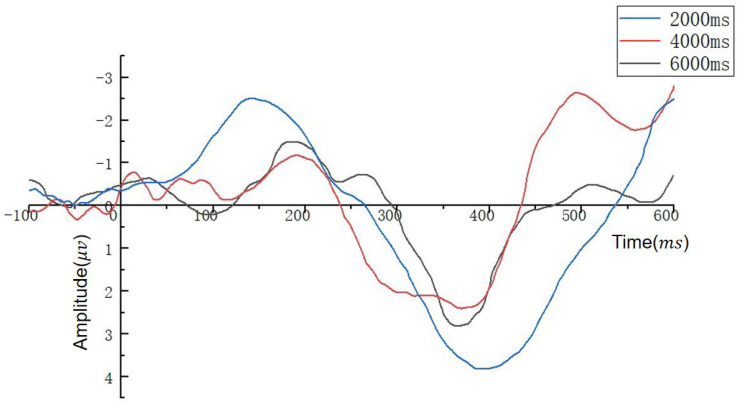
P300 time pressure waveform graph.

EEG signals were analyzed under different temporal pressures as follows: repeated-measures ANOVA was performed on the mean amplitude of 3 (i.e., 6000, 4000, and 2000 ms) × 7 (7 electrodes: PO4, P4, POz, Pz, CPz, PO3, and P3) from 250 to 750 ms. The ANOVA showed that the mean amplitude at electrode positions from 250 to 750 ms differed significantly between time pressure and electrodes because all *p*-values were less than 0.05. Therefore, an ANOVA was performed on the mean amplitude under time pressure in three brain regions (left, middle, and right), with the seven electrodes divided into right (PO4 and P4), middle (POz, Pz, and CPz), and left (PO3 and P3) groups. ANOVA results showed a significant main effect of the mean amplitude of brain regions under different temporal pressures (2000 ms: F(4,16) = 2.634, *p* = 0.045; 4000 ms: F(4,16) = 5.135, *p* = 0.014; 6000 ms: F(4,16) = 10.940, *p* = 0.012). Paired-sample tests were performed for the right, middle, and left mean amplitudes under different time pressures; if significant differences existed in any region, further t-tests were performed for the mean electrode amplitudes contained in each region to identify electrodes with significant differences under the three investigated time pressures. The results revealed a significant difference in the mean amplitude of Pz (*p* = 0.018). Therefore, the Pz potentials in the parietal cortex at different temporal pressures were of most interest; the P300 waveforms of the Pz electrodes are shown in Figure 8. The results of the comparison of the waveform amplitude and latency of the three groups of subjects on different tasks were as follows: 2000 ms > 4000 ms > 6000 ms.

The EEG data analysis procedure for different tasks at under same time pressure was the same as described above. The results revealed no significant difference in the mean amplitude of the electrodes between the graphical scalars under the two investigated time pressures, except for Pz. The P300 wave amplitude and latency under 4000 ms time pressure for the Pz electrode are shown in Table 7. The mean P300 wave amplitude and latency of subjects in the imagery icon group when completing the speed task were significantly lower than those for the threat and height tasks, and the wave amplitude and latency of the height task were the highest. The mean P300 wave amplitude and latency of subjects in the stereotype group when completing the threat task were much lower than those for the speed and height tasks, and the wave amplitude and latency of the speed task were the highest. The P300 mean wave amplitude and latency of subjects in the expert knowledge group when completing the threat task were significantly lower than those for speed and height tasks, whereas the speed and height tasks did not differ significantly.

### 4.2. Analysis of Experimental Data under Information Pressure Conditions

#### 4.2.1. Behavioral Data Analysis

As in the time stress condition experiment, the experimental data in the information stress condition included iconic memory recognition accuracy (ACC) and reaction time (RT).

Kruskal-Wallis tests were conducted on the correctness rates under different information stress conditions in the same group. The correctness rates of subjects completing the task in the imagery schema group, stereotype group, and expert intuition group differed significantly (*p* = 0.021; *p* = 0.013; *p* = 0.034), and post hoc multiple comparison corrections were performed; the results of the two comparisons differed significantly. Kruskal–Wallis tests were conducted on the accuracy rates of different groups under the same information pressure conditions. There were no significant differences in the correct rates of subjects completing the three types of tasks in the imagery schema, stereotype, and expert intuition groups under the single information pressure condition. Significant differences were found under both the two-information stress and three-information stress conditions (*p* = 0.013; *p* = 0.022), and post hoc multiple comparison corrections were performed. The results of the two comparisons were significantly different.

**Figure 9:**
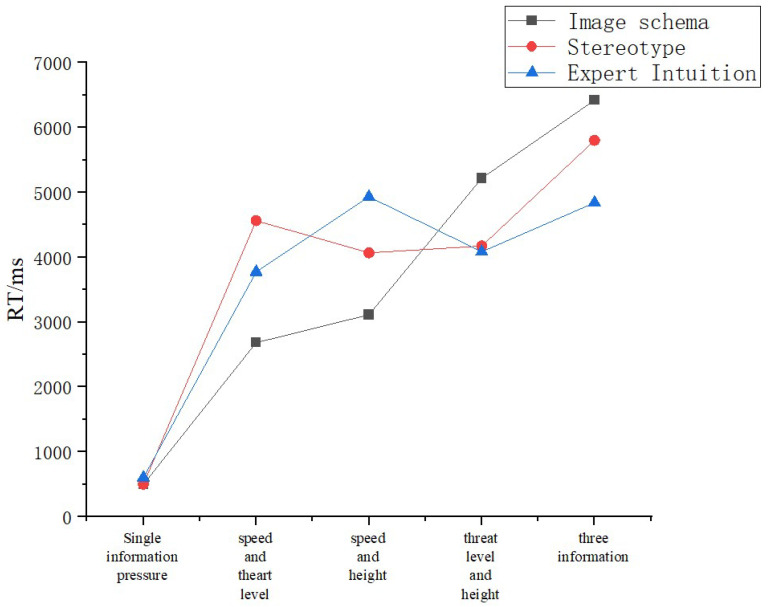
RTs.

Reaction times are shown in Figure 9. A one-way ANOVA was performed on reaction times between groups for the same task, and the results showed significant differences between all groups. A one-way ANOVA was conducted on the reaction times of subjects under the same information pressure in the same group, and the results showed no significant differences in the two-task information pressure condition for the expert knowledge group only. As shown in Figure 9, the imagery schema group performed better under the single-task and dual-task conditions, but the total time spent on the three-task condition increased. The stereotype group spent more time than the imagery schema group on the speed and threat tasks.

#### 4.2.2. EEG Data Analysis

For EEG data processing process under the same temporal pressure condition, seven electrodes were selected were selected in the parieto-occipitotemporal joint cortex (P3, CPz, Pz, POz, PO3, P4, and PO4), and EEG signals were analyzed under different information pressure conditions as follows: for 3 (i.e., single-task, dual-task, and triple-task) × 7 (7 electrodes: PO4, P4, POz, Pz, CPz, PO3, and P3) 250~, an average amplitude of 750 ms was subjected to repeated measurement ANOVA. The ANOVA results showed that the mean amplitudes of electrode positions at 250~750 ms differed significantly between temporal pressure and electrodes, as all *p*-values were less than 0.05. Therefore, an ANOVA was performed on the mean amplitudes of information pressure and three brain regions (left, middle, and right), the results of which showed that the mean amplitudes of brain regions had a significant main effect across information pressure conditions (single task: F = 8.628, *p* = 0.102; dual task: F = 2.705, *p* = 0.238; triple task: F = 4.30, *p* = 0.021). Paired-sample tests were performed on the right, middle, and left mean amplitudes for different temporal pressures; if significant differences existed in any region, a further *t*-test was performed on the mean electrode amplitudes contained in each region to identify electrodes with significant differences under the three information pressure conditions. The results revealed a significant difference in the mean amplitude of Pz (*p* = 0.008). Therefore, the Pz potential in the parietal cortex under different information stress conditions was of most interest, as shown in Table 8. (1) Overall, the order of P300 wave amplitude and latency under the three information stress conditions, from small to large, was: single information < double information < triple information. (2) Subjects in the imagery illustration group exhibited the lowest P300 wave amplitude and the shortest latency during the speed threat level task; the opposite was true for the threat level and height tasks. (3) Subjects in the stereotype group exhibited the lowest P300 wave amplitude and the shortest latency period when completing the height threat level task; the opposite was true for the speed and height tasks. (4) Subjects in the expert knowledge group exhibited the lowest P300 wave amplitude and the shortest latency period when completing the speed and height tasks.

## 5. Discussion

We conducted two kinds of experiments in the present study to investigate the research questions. The results of the time stress experiment show that the greater the time pressure, the higher the cognitive load and the higher the error rate. The results of the information stress experiment show that with an increased number of tasks, the cognitive load of subjects increased, and the cognitive load associated different combinations of tasks with the same total number of tasks differed significantly.

In terms of behavior data, people are very sensitive to the color and brightness of the target. In the time pressure experiment, the behavioral data showed that the subjects were faster and more accurate in completing the two search tasks (brightness represents the speed in the image representation group, and color represents the threat in the stereotype group), far exceeding performance on other tasks. This is consistent with the results reported by Beatriz under low brightness contrast; the addition of color information can shorten RT, and RTs are be strongly influenced by stimulus size and adaptive brightness contrast [32]. Eye data show that subjects do not require a long search process when searching for these two attributes, and they can obtain most of the target information in one gaze, supporting the advantages of color and brightness. Moreover, scanning path data indicate that when subjects in the image illustration group searched for the speed target, the gaze point always appeared on or near the brightest target first, demonstrating that bright icons attract more attention, enabling subjects to find quickly among many icons. Prior knowledge of the physical characteristics of the observer are among the most important factors influencing effective searches, significantly improving the detection performance [33]. This may explain why subjects in the image representation group were efficient in searching for speed targets. In the stereotype group, when the subjects completed the threat search task, the scanning path rarely passed through the icon required by the task, although the subjects were able to complete the task quickly and accurately. This result supports the conclusion reported by Schall and Hanes [34]; when a color is contained in the sought target, the neurons of the positive visual field (FEFs) react more strongly to it Schall and Hanes [34] than the color is associated with a distractor. This shows that people’s perception of color may be more sensitive than that of brightness and that subjects’ cognitive load may be reduced when performing color search tasks. Expert searchers usually cite target knowledge as a key factor in reducing errors and successfully finding targets. In the expert knowledge group, we found that the performance of the subjects was similar in completing the speed and height search tasks was, indicating that cognitive load does not change with respect to the representation of the same type of features. According to EEG data, the amplitude and latency of the three groups of subjects in different tasks were: 2000 ms > 4000 ms > 6000 ms. These results demonstrate that the subjects required more psychological and cognitive resources and bore a heavier memory load under the 2000 ms time pressure condition, which is also consistent with the results of previous research on the positive correlation between psychological resources in the two-task experiment [34]. The amplitude of P300 of the image group on the speed task was lower than that of the other two tasks, indicating that the subjects’ cognitive load on speed task was low. The latency was also minimal for the speed task, indicating that the processing order of image brightness occurs before the size and filling degree of graphics. In the stereotype group, the P300 amplitude and latency of threat task were lower than those for the other two tasks, indicating that the subjects’ processing order of color occurred to picture shape and semantics. In the knowledge group, the amplitude and latency for the altitude and speed tasks were similar and lower than those for the threat task, indicating that the subject perceived the quantity change of features faster than the appearance change, independent of the meaning represented by the features. In the information pressure condition experiment, the same group exhibited significant differences in accuracy and reaction time under different information pressures, indicating that information pressure has a considerable influence on reaction time and accuracy. In terms of reaction time, subjects in all three groups reacted faster when the information pressure was lower.

Under the condition of double information pressure, the subjects in the image representation group completed the double tasks with speed attributes faster. When the stereotype group completed the task combination with threat attributes, the reaction time was shorter than that of the single task, and the accuracy rate also improved, confirming the conclusion reported Schall and Hanes. Under the condition of three information pressures, the expert knowledge group exhibited lower time consumption than that of the other two groups, possibly because the design of the three target attributes of the expert knowledge group were similar, and all of them used different shapes or lines to express the target attributes, so the task understanding time was short, making it easier to combine the three tasks in the cognitive process. Analysis of PZ electrode P300 amplitude and latency under different information pressure conditions showed that the cognitive load associated with the speed threat task was lowest in the image representation group under the condition of double information pressure. In the stereotype group, the subjects’ cognitive coincidence was lowest in completing the height and threat tasks. In the knowledge group, the cognitive conformity of the subjects when completing the height and speed tasks was lower than when completing the other two task combinations. On the whole, the greater the information pressure, the more difficult it was for the subjects to complete the task. Under the condition of three information pressures, the amplitude and latency of P300 in the expert intuition group were lowest, indicating that in the expert intuition group, the subjects were able to quickly combine various information features, with minimal increase in cognitive coincidence, surpassing the image representation group and the stereotype group only when the information pressure reached when the information pressure reached the third level.

In summary, the experimental results show that different intuitive design methods affect the cognitive difficulty of tasks, mainly with respect to the source of knowledge that causes intuition. The closer the design is to physical reality, the faster the intuitive cognitive speed. However, when the number of attributes to be observed increases, the closer the design is to physical reality, greater the cognitive pressure. Therefore, on a limited display interface, icons should be designed according to the actual needs to achieve an optimal intuitive design effect.

## 6. Summary

Modern warfare is demanding on people; the ability to selecting a desired target among a large amount of information in a short period of time is a necessary capability for commanders and equipment operators. However, human conscious processing ability is limited, so it is important to make reasonable use of unconscious processing ability. In this study, we explored the respective advantages and cognitive mechanisms of different intuitive design approaches under time and information pressure conditions. Furthermore, we investigated intuitive design approaches for a visual search task on a radar display interface.

The results of this study can be summarized as follows: (1) When representing the attributes of a target, image icons are most suitable for expressing the motion attributes of targets, such as speed and height, whereas stereotypes are most suitable for expressing fixed attributes, such as target threat. (2) Tasks with high time pressure are more difficult, leading to higher error rates; the reaction time of a single task should be no less than 4000 ms. (3) When multiple attributes of the target need to be observed at the same time, the same type of expression should be used. For example, one kind of graph is used to represent one attribute of the target, and another other kind of graph is used to represent another attribute of the target, as cognitive load is reduced when dealing with multiple pieces of information of the same kind. However, an excessive number of expressions of the same kind can increase memory load. (4) Rational use of color to represent the target attributes can effectively reduce the cognitive load of people searching for the target.

The research results reported herein can be used to inform design principles for intuitive design of icons for radar search interfaces. Using these principles to design radar interface icons can improve search efficiency, reduce cognitive load, and reduce mistakes under conditions of time and information pressure.

## Figures and Tables

**Table 1:** Example of speed display design.

Design Method	High Speed	Medium Speed	Low Speed
Image schema	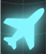	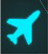	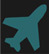
Stereotype	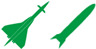	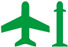	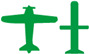
Expert Intuition	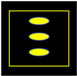	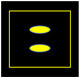	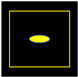

**Table 2:** Image schema list.

Category	Image Schema
Basic mode	Matter, object
space	Up and down, left and right, near and far, front and back, center and periphery, contact, path, proportion
control	Container, access, contents, empty, surface
consistent	matching
diversity	Merging, collecting, dividing, partial whole, counting quality, linking
process	Superposition, iteration, and circulation
force	Transfer, reaction force, constraint elimination, resistance, attraction, force, blockage, balance, momentum, potential energyTransfer, reaction force, constraint elimination, resistance, attraction, force, blockage, balance, momentum, potential energy
attribute	Light, dark and bright, big to small, warm and cold, strong and weak, smooth and rough, straight.

**Table 3:** Example of height display design.

Design Method	High Altitude	Medium Altitude	Low Altitude
Image schema	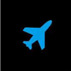	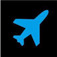	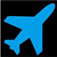
Stereotype	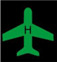	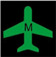	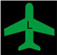
Expert Intuition	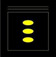	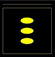	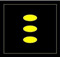

**Table 4:** Example of threat level display design.

Design Method	High Threat	Medium Threat	Low Threat
Image schema	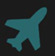	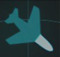	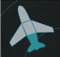
Stereotype	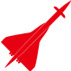	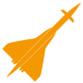	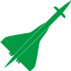
Expert Intuition	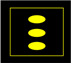	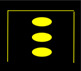	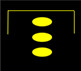

**Table 5:** Types of experimental interfaces.

Image Schema	Stereotype	Expert Intuition
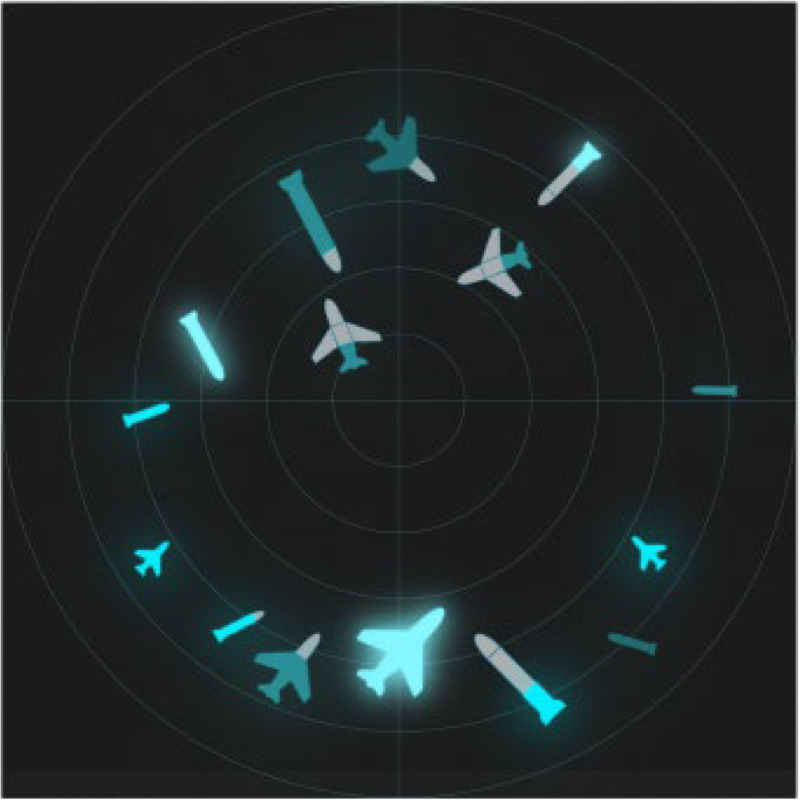	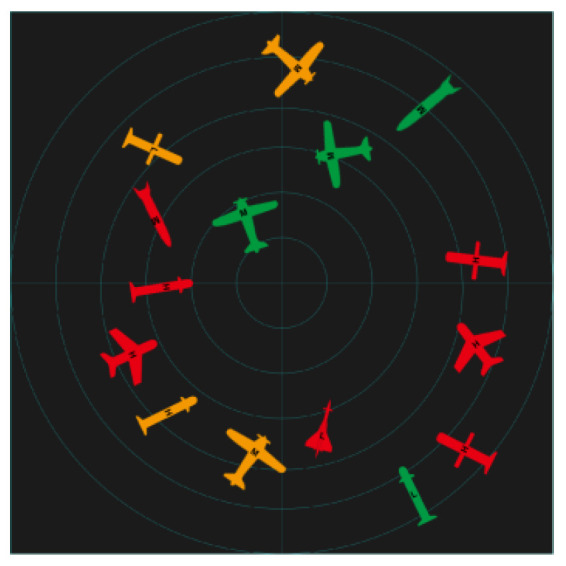	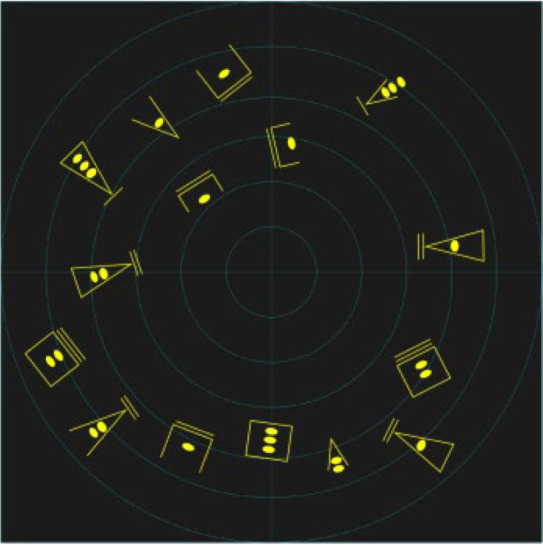

**Table 6:** Single attributes possessed by the sample icons.

Height Attribute	Threat Attribute	Speed Attribute
High altitude	High Threat	High Speed
Mid altitude	Mid threat	Mid speed
Low altitude	Low threat	Low speed

**Table 7:** P300 amplitude and latency for different tasks.

Task	Wave Amplitude (Unit: μv)			Incubation Period (Unit: ms)		
	Image Schema	Stereotypes	Expert Intuition	Image Schema	Stereotypes	Expert Intuition
Speed	6.1	2.12	3.2	302	413	354
Altitude	7.35	4.36	2.37	421	543	354
Threat level	7.23	−2.63	2.34	346	290	391

**Table 8:** Average P300 amplitude and latency under different information pressure conditions.

Information Pressure Level	Wave Amplitude (Unit: μv)			Incubation Period (Unit: ms)		
	Image Schema	Stereotype	Expert Intuition	Image Schema	Stereotype	Expert Intuition
Single information	4.32	4.14	4.79	376	368	397
Speed and threat level	6.27	6.1	6.43	405	444	441
Speed and height	6.19	7.97	6.39	417	551	421
Threat level and height	6.51	5.93	6.22	518	412	455
Three tasks	8.14	9.19	6.95	663	611	546

## Data Availability

The raw data supporting the conclusions of this article will be made available by the authors, without undue reservation.
